# Diagnostic and Prognostic Non-Invasive Markers in Bladder Cancer

**DOI:** 10.3390/diagnostics16131948

**Published:** 2026-06-23

**Authors:** Ki Choon Sim, Min Ju Kim, Deuk Jae Sung, Beom Jin Park, Na Yeon Han, Yeo Eun Han, Seung Ha Cha

**Affiliations:** Department of Radiology, Korea University Anam Hospital, Korea University College of Medicine, 73 Goryeodae-ro, Seongbuk-gu, Seoul 02841, Republic of Korea; kspringsim@korea.ac.kr (K.C.S.); urorad@korea.ac.kr (D.J.S.); radiolbj226@gmail.com (B.J.P.); mammos@korea.ac.kr (N.Y.H.); yeonny0714@korea.ac.kr (Y.E.H.); seunghacha@gmail.com (S.H.C.)

**Keywords:** bladder cancer, multiparametric MRI, VI-RADS, non-invasive biomarkers, urine cytology, liquid biopsy, radiomics, artificial intelligence, treatment response, biomarker

## Abstract

Bladder cancer is a common malignancy with high recurrence rates and significant morbidity, necessitating accurate diagnostic and prognostic tools. Although cystoscopy and transurethral resection of bladder tumor (TURBT) remain reference standards, these approaches are invasive and limited by sampling errors and understaging. Consequently, there is growing interest in non-invasive biomarkers, including urine-based assays, blood-based markers, and imaging-derived parameters. Among these biomarkers, multiparametric magnetic resonance imaging (mpMRI), particularly with the Vesical Imaging Reporting and Data System (VI-RADS), has emerged as a robust non-invasive imaging biomarker for local staging and risk stratification. Recent evidence suggests that mpMRI plays a role in predicting treatment response and recurrence, particularly in the context of neoadjuvant therapy. This review provides a comprehensive overview of current non-invasive diagnostic and prognostic biomarkers in bladder cancer, with a particular emphasis on imaging biomarkers. We discuss their clinical utility, limitations, and future integration into multimodal decision-making frameworks.

## 1. Introduction

Bladder cancer is one of the most common malignancies worldwide, with more than 614,000 new cases diagnosed annually [1]. It is characterized by a high rate of recurrence, particularly in non–muscle-invasive disease, where recurrence occurs in up to 70% of patients during follow-up [2,3]. The global burden of bladder cancer is projected to increase substantially, with its annual incidence and mortality estimated to rise by 73% and 87%, respectively, by 2040, underscoring the urgent need for improved diagnostic and therapeutic strategies [4]. Urothelial carcinoma accounts for approximately 90–95% of all cases of bladder cancer [5,6]. Bladder cancer is broadly classified into non–muscle-invasive bladder cancer (NMIBC) and muscle-invasive bladder cancer (MIBC). The presence or absence of detrusor muscle invasion is the pivotal landmark that distinguishes these two types, because this landmark dictates the transition from bladder-preserving strategies to more aggressive definitive treatments [2,7]. These fundamentally different prognostic and therapeutic implications underscore the clinical necessity for accurate T-staging [6,8]. Cystoscopy and transurethral resection of bladder tumor (TURBT) remain the gold standard for diagnosis and staging. However, TURBT is associated with notable limitations, including sampling error and understaging in up to 50% of cases [9,10,11,12], underscoring the need for reliable, non-invasive biomarkers that can improve diagnostic accuracy and guide clinical decision-making.

Recent advances in molecular diagnostics have expanded the landscape of non-invasive biomarkers, particularly through urine-based liquid-biopsy approaches that enable the detection of tumor-derived DNA and genomic alterations with encouraging diagnostic performance [13]. These molecular assays, including mutation-based panels targeting genes such as *TERT*, *FGFR3*, and *PIK3CA*, have demonstrated improved sensitivity compared with conventional urine cytology, although their role in clinical practice continues to develop [13,14,15,16].

In addition to molecular and urine-based approaches, imaging has emerged as a distinct and increasingly important category of non-invasive biomarkers in bladder cancer. In particular, multiparametric magnetic resonance imaging (mpMRI) provides anatomical and functional information, enabling accurate tumor characterization, staging, and prognostic assessment. Since the introduction of the Vesical Imaging-Reporting and Data System (VI-RADS) in 2018, which established a standardized framework for bladder MRI interpretation, research exploring imaging-based biomarkers and their clinical utility in bladder cancer has increased substantially [5,6,8,17].

In this narrative review, we aimed to comprehensively summarize current non-invasive biomarkers in bladder cancer, with a particular emphasis on the evolving role of imaging—especially multiparametric MRI—as a diagnostic and prognostic tool. We further explore the potential integration of these biomarkers into clinical workflows to improve risk stratification and guide treatment decisions.

## 2. Methods

This narrative review was conducted to summarize current evidence regarding non-invasive diagnostic and prognostic biomarkers in bladder cancer, with a particular focus on imaging biomarkers and multiparametric MRI.

A literature search was primarily performed using PubMed and Google Scholar databases for studies published in English up to March 2026. Relevant articles were selected based on their clinical relevance, methodological quality, and contribution to the evolving field of bladder cancer biomarkers, including urine-, blood-, and imaging-based approaches.

In addition, this review incorporates the clinical perspectives and practical experience of the authors, including radiologists with expertise in genitourinary imaging and bladder MRI, particularly within the Korean clinical setting. In evaluating the available evidence, priority was given to findings from meta-analyses, prospective multicenter studies, and randomized controlled trials, whereas single-center retrospective studies were considered supplementary sources.

As this study was designed as a narrative review, no formal systematic review protocol or PRISMA-guided study selection process was applied.

## 3. Current Diagnostic Standards and Their Limitations

### 3.1. Traditional Urine Cytology

Alongside cystoscopy, voided urine cytology has long been the standard non-invasive adjunct for bladder cancer detection and surveillance. Its primary advantage lies in its high specificity (frequently exceeding 90%), particularly for high-grade urothelial carcinoma and carcinoma in situ (CIS) [5,8]. Urine cytology is a simple, cost-effective, and completely non-invasive method that relies on the microscopic evaluation of exfoliated malignant cells in the urine. These characteristics make it well-suited for repeated testing, enabling its use as a serial surveillance tool throughout the long-term follow-up of patients with bladder cancer.

However, urine cytology is hampered by critically low sensitivity, especially for low-grade tumors, where it often fails to provide a diagnosis owing to the well-differentiated nature of cells [5,18]. Additionally, the interpretation of its results is highly dependent on the expertise of the pathologist; results can be obscured by low cellularity, urinary tract infections, or intravesical inflammation following Bacillus Calmette-Guérin (BCG) therapy [18,19]. These diagnostic gaps—especially the poor sensitivity for low-risk disease—have traditionally necessitated the use of more invasive procedures for definitive staging [8].

Advancements in urinary cytology have emphasized a multidimensional approach to overcome the aforementioned limited sensitivity. The Paris System for Reporting Urinary Cytology, first introduced in 2016 and subsequently updated in 2022, provides a standardized reporting framework with a primary focus on the detection of high-grade urothelial carcinoma [19,20,21]. Beyond standardized reporting, the integration of artificial intelligence (AI)-driven digital pathology has reduced inter-observer variability and enhanced detection rates in equivocal cases [22]. These cytomorphological refinements, when coupled with molecular urine assays, have helped to establish a more robust, non-invasive diagnostic framework for bladder cancer [13].

### 3.2. TURBT

Owing to the limitations of non-invasive tools, such as cytology, TURBT remains the cornerstone of bladder cancer management. It offers several distinct advantages; primarily, it provides a definitive histopathologic diagnosis, which is essential for confirming urothelial carcinoma and identifying histological variants [5,6]. Tissue sampling during TURBT also enables precise tumor grading and pathologic staging, which are indispensable for risk stratification. In many patients with NMIBC, the procedure is not only diagnostic but also therapeutic, as it can achieve the complete resection of all visible lesions [2,9]. Ultimately, by establishing the presence or absence of muscularis propria invasion, TURBT plays a central role in determining the trajectory of subsequent management, including intravesical therapy, radical cystectomy, or multimodal bladder-preserving strategies [2,3,7].

Despite remaining the century-old ‘gold standard’ for bladder cancer diagnosis and staging, TURBT has notable disadvantages. A principal limitation is that the muscularis propria is not always included in the resection specimen, inherently reducing staging accuracy [11]. Consequently, residual tumor and understaging (observed in up to 50% of cases) are frequent occurrences after the initial procedure, often necessitating a second-look TURBT [11,12]. As an operative procedure, TURBT is invasive and carries the risk of complications such as bladder perforation, hematuria, and other procedure-related morbidities [23]. Furthermore, the reliability of the procedure is limited by inter-observer variability in pathologic assessment, meaning the final staging may not always be fully reproducible [24]. These limitations are particularly consequential in patients with potentially muscle-invasive disease, in whom inaccurate initial staging may lead to a delay in definitive treatment, consequently negatively impacting long-term survival [11,12].

## 4. Urine-Based Biomarkers

Urine-based biomarkers represent an intuitive and highly accessible approach for detecting NMIBC. Given that urine is in direct and continuous contact with the urothelium, it serves as a rich reservoir for exfoliated tumor cells, secreted proteins, and tumor-derived molecular products. The clinical aspiration for these markers is to provide a “liquid biopsy” that can either replace or significantly reduce the frequency of invasive cystoscopic surveillance.

### 4.1. Protein-Based Markers

Historically, protein-based markers have been the most widely investigated adjuncts to cytology. Assays for Nuclear Matrix Protein 22 (NMP22) and Bladder Tumor Antigen (BTA; available as BTA-stat and BTA-TRAK) are among the few US Food and Drug Administration (FDA)-approved urinary assays [25]. These markers generally offer superior sensitivity compared with traditional urine cytology, particularly in the detection of low-grade tumors. However, their clinical utility is severely hampered by suboptimal specificity. False-positive results may occur in clinical scenarios such as urinary tract infections, hematuria, urolithiasis, or intravesical inflammation, which are conditions commonly encountered during bladder cancer surveillance [25,26]. Consequently, although these markers provide high negative predictive value, their propensity for false positive results limits their role to a supportive one rather than as a definitive diagnostic tool [2]. Contemporary clinical guidelines do not recommend replacing cystoscopy with these urinary biomarkers owing to their limited specificity and inconsistent performance across clinical settings [3].

Reviews have further emphasized that, despite their historical significance, assays for NMP22 and BTA have largely been superseded by advanced molecular assays with improved diagnostic accuracy, reinforcing their current role as adjunctive rather than standalone diagnostic tools [27].

### 4.2. Genomic and Molecular Markers

Advances in molecular diagnostics have shifted the focus toward genomic alterations that drive urothelial carcinogenesis. These emerging biomarkers target specific DNA or RNA changes with high precision.

• Gene mutations: Panels targeting *FGFR3*, *TERT* promoter, and *PIK3CA* mutations have demonstrated favorable diagnostic performance, often identifying malignancy before it becomes visible on cystoscopy [13,14,15,16]. Of the genomic targets described above, FGFR3 merits particular attention as both a diagnostic and therapeutic biomarker in NMIBC. Activating mutations or fusions are detected in up to 75% of non-muscle-invasive tumors, making it a dominant molecular feature of the luminal papillary subtype [15]. Beyond its diagnostic implications, FGFR3 alteration status may also inform therapeutic decision-making. Emerging evidence suggests that FGFR-targeted therapies can provide favorable clinical outcomes in selected patients with FGFR3-altered NMIBC, with treatment response potentially influenced by the specific alteration subtype, including FGFR3-TACC3 fusions and point mutations such as S249C [16]. These findings underscore the clinical value of comprehensive FGFR3 molecular characterization for precision therapeutic stratification in bladder cancer.
• Epigenetic markers: DNA methylation panels (e.g., *Bladder EpiCheck*) offer high sensitivity and have shown particular value for NMIBC surveillance [28].
• Liquid-biopsy approaches: Digital droplet polymerase chain reaction (ddPCR) and next-generation sequencing (NGS) of urinary sediment enable the detection of minute quantities of tumor-derived DNA [13]. Additionally, urinary tumor DNA (utDNA) has emerged as a valuable biomarker for detecting minimal residual disease following radical cystectomy. Evidence suggests that utDNA-based assays can identify residual or recurrent disease at an early stage, potentially preceding conventional radiologic or cystoscopic findings [29].

Despite their high diagnostic accuracy in clinical trials, these molecular assays have not yet achieved widespread clinical adoption owing to high costs, lack of standardized reporting, and the requirement for specialized laboratory infrastructure.

### 4.3. Limitations and Current Status

Despite substantial technological strides, urinary biomarkers currently cannot replace cystoscopy as the gold standard. Their primary role remains adjunctive, serving as tools for risk stratification or potentially reducing the frequency of invasive procedures in selected low-risk patients. A notable confounding factor is the molecular field effect, which is characterized by genetic alterations in morphologically normal-appearing urothelium [30,31]. This phenomenon can lead to ‘anticipatory positive’ results, causing diagnostic uncertainty when molecular markers indicate malignancy that is not yet visible via cystoscopy. In addition, pre-analytical variables, including urine concentration and sample collection timing, may affect test reproducibility. Therefore, integrating these biomarkers into a multimodal decision-making framework combining molecular data with advanced imaging may represent a promising strategy for precision management in bladder cancer.

Although several urinary biomarkers have demonstrated encouraging diagnostic performance in early studies, their performance in large-scale real-world clinical settings has often been less consistent. Many initial studies were conducted in relatively small or highly selected patient cohorts, potentially overestimating diagnostic accuracy because of spectrum bias and enriched disease prevalence. In addition, heterogeneity in assay platforms, patient populations, and diagnostic thresholds has limited cross-study comparability and slowed broader clinical adoption. The relatively high cost of proprietary molecular assays, limited reimbursement, and the need for specialized laboratory infrastructure further restrict their cost-effectiveness and widespread implementation, particularly in resource-limited healthcare settings. Therefore, urinary molecular assays are currently best considered adjunctive tools within risk-adapted and multimodal surveillance strategies rather than standalone replacements for conventional cystoscopic surveillance [3,27].

## 5. Blood-Based Biomarkers

Although urine-based assays provide direct insights into the local tumor environment, blood-based biomarkers—often referred to as systemic liquid biopsies—offer a broader perspective on tumor burden, metastatic potential, and therapeutic response. These approaches are particularly valuable in patients with muscle-invasive or advanced diseases, in whom systemic dissemination is a major concern.

### 5.1. Circulating Tumor DNA (ctDNA)

ctDNA comprises fragmented DNA released into the bloodstream by apoptotic or necrotic tumor cells. Recent longitudinal studies have demonstrated that ctDNA is a highly sensitive biomarker for detecting minimal residual disease following radical cystectomy [32]. The presence of ctDNA in the perioperative period is strongly associated with an increased risk of recurrence and poorer disease-free survival.

Moreover, dynamic changes in ctDNA levels may serve as an early indicator of treatment response to neoadjuvant or adjuvant therapy and can precede radiologic evidence in selective clinical settings [32,33].

### 5.2. Circulating Tumor Cells (CTCs)

CTCs are intact malignant cells that have detached from the primary tumor and entered the peripheral bloodstream. In bladder cancer, particularly in non–muscle-invasive disease, CTC detection has been investigated as a prognostic biomarker. Higher CTC counts have been associated with adverse pathological features, including higher tumor stage and increased risk of recurrence or progression [34].

Although technical challenges in CTC isolation and enrichment remain, emerging technologies such as microfluidic platforms and molecular profiling of CTCs (e.g., PD-L1 expression) may enable personalized treatment strategies, including immunotherapy selection.

### 5.3. Inflammatory and Systemic Markers

In addition to tumor-derived genetic material, systemic inflammatory markers have gained attention as cost-effective prognostic indicators. The neutrophil-to-lymphocyte ratio (NLR) reflects the host inflammatory response to malignancy and has been associated with disease progression and oncologic outcomes in bladder cancer. Elevated pre-treatment NLR has been linked to a worse prognosis in NMIBC and MIBC [35,36].

Although these markers lack tumor specificity, their accessibility and ease of integration into routine clinical practice make them useful for baseline risk stratification.

### 5.4. Limitations of Blood-Based Approaches

Despite their potential, blood-based biomarkers face several limitations that currently restrict their use in routine clinical practice, particularly in localized bladder cancer. The concentration of ctDNA and CTCs in early-stage NMIBC is often extremely low, resulting in limited sensitivity compared with urine-based assays.

In addition, the lack of standardized methodologies for sample processing and analysis, along with the high cost associated with high-depth sequencing technologies, presents significant barriers to widespread implementation. Therefore, at present, blood-based biomarkers are primarily utilized as prognostic tools for aggressive or advanced disease rather than as primary diagnostic modalities for early-stage bladder cancer. Furthermore, despite promising results from early investigational studies, the clinical translation of blood-based biomarkers has been slower than anticipated because of limited sensitivity in localized disease, inter-study heterogeneity, and the lack of large prospective validation studies.

Table 1 provides a comparative summary of the key urine-based, blood-based, and imaging-based non-invasive biomarkers discussed in this review, highlighting their respective clinical utilities, advantages, limitations, level of evidence, cost, and clinical readiness. Of note, key clinical evidence for ctDNA has been largely derived from industry-sponsored immunotherapy trials (e.g., IMvigor series, Roche/Genentech), and CTC studies are constrained by dependence on a single proprietary platform (CellSearch; Menarini Silicon Biosystems); both factors should be considered when evaluating the independence of the reported validation data. In contrast, NLR, mpMRI, and bpMRI are supported by independently funded academic evidence without commercial sponsorship concerns.

## 6. Imaging Biomarkers: The Central Role of mpMRI

The diagnostic paradigm for bladder cancer has evolved from purely anatomical assessment toward increasing incorporation of functional characterization. mpMRI has emerged as a promising non-invasive imaging biomarker that may help bridge the gap between clinical suspicion and pathological confirmation. By integrating morphological, cellular, and vascular data, mpMRI provides a multidimensional assessment of tumor characteristics of the tumor that may complement conventional imaging modalities, such as computed tomography (CT) or ultrasound.

### 6.1. Biological Basis of mpMRI as a Multimodal Biomarker

The strength of mpMRI lies in its ability to simultaneously interrogate different biological properties of the tumor tissue. For a diagnostic tool to be considered a “biomarker,” it must provide reproducible and objective indications of biological processes. mpMRI achieves this through three core pillars.

#### 6.1.1. T2-Weighted Imaging (T2WI): Morphological Characterization

T2WI remains the backbone of bladder MRI, providing the highest spatial resolution for anatomical staging. It allows for the clear visualization of the low-signal muscularis propria, which is a key landmark in bladder cancer staging. As a biomarker, T2WI assesses the structural integrity of the bladder wall. A continuous, dark line of the detrusor muscle indicates localized disease; an interruption of this line suggests muscle invasion. Nevertheless, T2WI alone is often limited by post-TURBT changes, such as edema or fibrosis, which can mimic tumor invasion and lead to potential overstaging [5,17,37].

#### 6.1.2. Diffusion-Weighted Imaging (DWI): A Surrogate for Cellularity

DWI is a functional sequence that measures the Brownian motion of water molecules within the tissue interstitium. In malignant tissues, the high nuclear-to-cytoplasmic ratio and dense cellular packing restrict the movement of water. Consequently, DWI serves as a biomarker for cellularity. The quantitative component of this sequence, the apparent diffusion coefficient (ADC) map, allows for a more objective assessment. High-grade urothelial carcinomas typically demonstrate significantly lower ADC values compared with low-grade tumors or inflammatory tissues [38]. Studies have suggested a correlation between ADC values and the Ki-67 proliferation index, cementing DWI’s role as a surrogate for tumor aggressiveness [39].

#### 6.1.3. Dynamic Contrast-Enhanced (DCE) Imaging: Mapping Vascularity

DCE imaging evaluates the microvascular environment of the tumor. Angiogenesis is a prerequisite for tumor growth and invasion; bladder cancers characteristically exhibit early and intense enhancement compared with the normal bladder wall. As an imaging biomarker for vascularity, DCE is particularly sensitive in identifying the early enhancement of the inner layer of the bladder wall, which may precede visible morphological changes on T2WI. This feature is especially useful in distinguishing a tumor stalk or sessile tumor base from the underlying detrusor muscle [17,40].

### 6.2. VI-RADS: Elevating Imaging to a Standardized Biomarker

The introduction of the VI-RADS in 2018, an important development in the field, contributed to more standardized interpretation of bladder MRI into a standardized and reproducible imaging assessment system. By providing a unified language for radiologists and urologists, VI-RADS has addressed the long-standing issue of inter-observer variability.

#### 6.2.1. Clinical Interpretation of the 5-Point Scale

**Figure 1:**
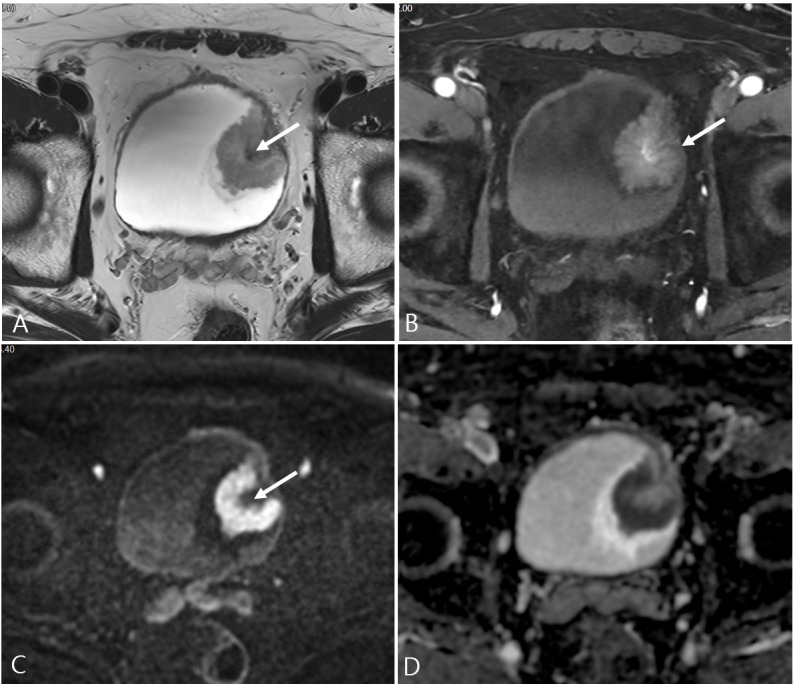
VI-RADS 2—Non-muscle-invasive bladder cancer. A 69-year-old male presenting with a 4.3-cm papillary mass in the left lateral wall of the bladder. (**A**) Axial T2-weighted imaging (T2WI) demonstrates a large papillary mass with an intact low-signal muscularis propria, showing a tumor stalk sign (arrow) and a suspected thickened inner layer, consistent with the involvement of the urothelium and lamina propria without muscle invasion. (**B**) Axial T1-weighted contrast-enhanced imaging (T1CE) shows enhancement confined to the tumor body, while the underlying muscularis propria maintains preserved low-signal intensity (arrow), indicating intact muscle layer integrity and supporting non-muscle-invasive disease. (**C**) High b-value diffusion-weighted imaging (DWI; b = 1000 s/mm^2^) demonstrates marked diffusion restriction within the tumor lesion, sparing the tumor stalk (arrow), which is suggestive of the inchworm sign. (**D**) The apparent diffusion coefficient (ADC) map confirms significantly reduced ADC values, reflecting high tumor cellularity. The overall imaging features are consistent with a VI-RADS score of 2, suggesting a non-muscle-invasive tumor confined to the mucosa or lamina propria. Subsequent transurethral resection confirmed pathological stage T1 urothelial carcinoma, consistent with the mpMRI-based VI-RADS assessment.

**Figure 2:**
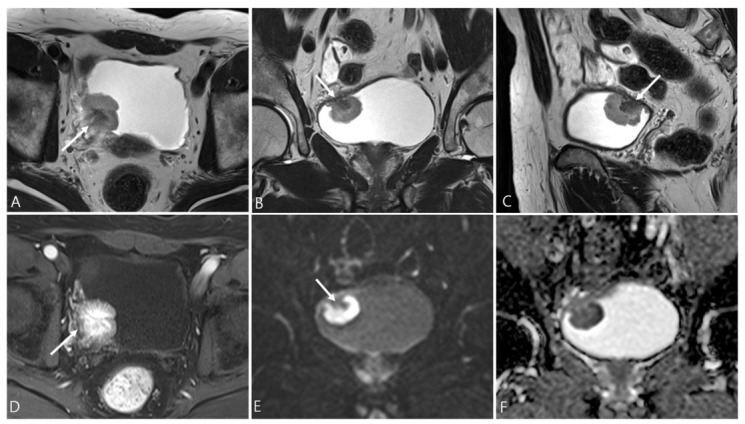
VI-RADS 2 or 3—Limitations of VI-RADS scoring: muscle invasion in a tumor with an apparent stalk at the bladder dome. A 69-year-old male presenting with a papillary mass at the right posterior dome of the bladder. (**A**) Axial T2-weighted imaging (T2WI) demonstrates a papillary mass at the right posterior dome; owing to the unfavorable anatomical location and suspected partial volume artifact, the accurate assessment of the tumor stalk and muscularis propria integrity was limited, leading to an equivocal VI-RADS 2 or 3 assessment on axial imaging alone. (**B**,**C**) Coronal and sagittal T2WI clearly delineate the tumor stalk sign with an intact low-signal muscularis propria; based on these multiplanar findings, the lesion was evaluated as VI-RADS 2. (**D**) Axial T1-weighted contrast-enhanced imaging (T1CE) shows early mucosal enhancement; however, evaluation of the inner layer and stalk remained suboptimal owing to the dome location. (**E**) Coronal diffusion-weighted imaging (DWI; b = 1000 s/mm^2^) demonstrates marked diffusion restriction within the tumor lesion, sparing the tumor stalk, which is suggestive of the inchworm sign. without evidence of extension into the muscularis propria. (**F**) Coronal apparent diffusion coefficient (ADC) map confirms significantly reduced ADC values within the lesion. Based on multiplanar mpMRI assessment, the overall imaging features were interpreted as VI-RADS 2, primarily guided by the tumor stalk sign on coronal and sagittal T2WI and the inchworm sign on DWI demonstrating an intact muscularis propria. However, subsequent TURBT unexpectedly confirmed muscle-invasive urothelial carcinoma. This case highlights a well-recognized pitfall of VI-RADS scoring: Kim et al. reported that approximately 11% of bladder tumors demonstrating a visible stalk on MRI (corresponding to a positive predictive value of 89% for the stalk sign) were ultimately confirmed as muscle-invasive on pathology [41], underscoring the inherent limitations of morphological assessment even with multiplanar imaging. Furthermore, although coronal and sagittal imaging are invaluable for dome lesions, they may paradoxically contribute to understaging when a partial volume artifact mimics an intact muscularis propria. Following neoadjuvant chemotherapy (NAC), partial cystectomy was performed. Preoperative mpMRI showed no evidence of residual tumor, and the final pathological staging confirmed a complete response (ypT0).

**Figure 3:**
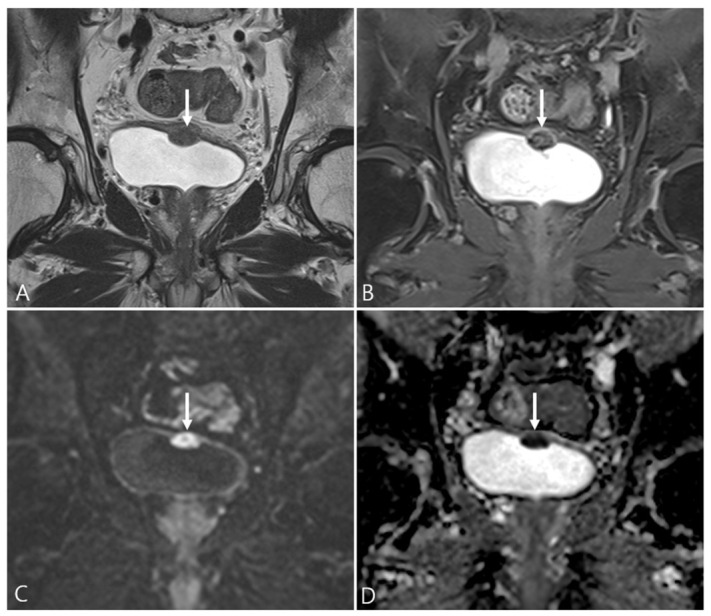
VI-RADS 4—Muscle-invasive bladder cancer at the bladder dome. An 83-year-old male presenting with a sessile mass at the bladder dome. Coronal images were preferentially acquired to optimize evaluation of the dome lesion, given its anatomical location at the superior aspect of the bladder. (**A**) Coronal T2-weighted imaging (T2WI) demonstrates an ovoid shaped mass at the bladder dome with disruption of the low-signal muscularis propria; the tumor stalk sign is not identified, raising concern for muscle invasion. (**B**) Coronal T1-weighted contrast-enhanced imaging (T1CE) shows early enhancement extending into the muscularis propria, supporting the likelihood of muscle-invasive disease. (**C**) High b-value diffusion-weighted imaging (DWI; b = 1000 s/mm^2^) demonstrates marked diffusion restriction within the tumor without the characteristic inchworm sign. (**D**) Apparent diffusion coefficient (ADC) map confirms significantly reduced ADC values, consistent with high tumor cellularity. The overall imaging features are consistent with a VI-RADS score of 4, indicating a high probability of muscle invasion. Subsequent TURBT confirmed pathological stage T2 urothelial carcinoma with concomitant carcinoma in situ (CIS). Given the patient’s refusal of radical cystectomy, concurrent chemoradiotherapy (CCRT) was administered as a bladder-preserving strategy, and the patient is currently under follow-up.

**Figure 4:**
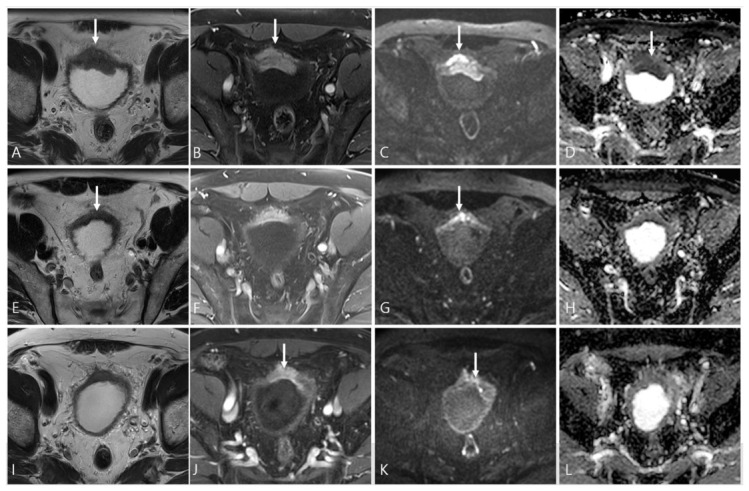
VI-RADS 5—Muscle-invasive bladder cancer with perivesical extension: treatment response monitoring with mpMRI. An 82-year-old male presenting with a bulky infiltrating mass at the anterior dome of the bladder. Sequential mpMRI was performed at baseline, during concurrent chemoradiotherapy (CCRT), and prior to radical cystectomy to monitor treatment response. Baseline MRI (**A**–**D**): (**A**) Axial T2-weighted imaging (T2WI) demonstrates bulky wall thickening with perivesical infiltration at the anterior dome, measuring up to 4.3 cm, with the disruption of the muscularis propria and ill-defined perivesical fat planes. (**B**) T1-weighted contrast-enhanced imaging (T1CE) shows the relatively homogeneous early enhancement of the lesion extending into the perivesical fat, consistent with extravesical tumor extension. (**C**) High b-value diffusion-weighted imaging (DWI; b = 1000 s/mm^2^) demonstrates marked diffusion restriction throughout the mass. (**D**) Apparent diffusion coefficient (ADC) map confirms significantly reduced ADC values. The overall baseline imaging features are consistent with a VI-RADS score of 5, indicating definitive muscle invasion with perivesical tumor extension (≥T3). Mid follow-up MRI after CCRT (**E**–**H**): (**E**) T2WI shows a reduction in the overall extent of the lesion following CCRT, with partial decrease in wall thickening. (**F**) T1CE demonstrates persistent significant enhancement within the residual lesion, raising strong concern for viable residual tumor. (**G**) DWI (b = 1000 s/mm^2^) reveals persistent diffusion restriction within the residual mass (arrow). (**H**) ADC map shows persistently reduced ADC values, further supporting the presence of residual viable tumor despite partial morphological response. Pre-operative MRI (**I**–**L**): (**I**) T2WI demonstrates further subtle reduction in lesion size; however, residual abnormal wall thickening with perivesical changes persists. (**J**) T1CE shows continued significant enhancement of the residual lesion (arrow). (**K**,**L**) DWI and ADC map reveal persistent moderate diffusion restriction, with a reduced extent compared to the prior study. Despite a partial morphological response on sequential imaging, the persistent significant enhancement and diffusion restriction were strongly suggestive of residual viable tumor. Based on the nacVI-RADS scoring system [43], these findings correspond to category 4 (partial response with persistent muscle-invasive disease). Subsequent radical cystectomy confirmed pathological stage ypT3b urothelial carcinoma, validating the mpMRI-based assessment of incomplete response to CCRT.

The VI-RADS score represents the probability of muscle invasion (MIBC) based on the integration of T2WI, DWI, and DCE findings:

• VI-RADS 1 and 2 (NMIBC Likely): These scores indicate that the tumor is likely confined to the mucosa or lamina propria. The muscularis propria appears intact across all sequences. In these cases, a high-quality TURBT may be sufficient as the primary treatment (Figure 1) [5,6,17].

• VI-RADS 3 (Equivocal): This category represents a “grey zone” wherein muscle invasion cannot be definitively ruled out, often owing to technical limitations or ambiguous findings. It necessitates a more cautious clinical approach, such as a formal re-resection or closer surveillance (Figure 2) [17].

• VI-RADS 4 and 5 (MIBC Likely): These scores indicate a high probability (Score 4, Figure 3) or definitive evidence (Score 5, Figure 4) of muscle invasion or extravesical extension. For these patients, the biomarker suggests a shift in management toward more aggressive strategies, including neoadjuvant chemotherapy (NAC) and radical cystectomy (Figure 4) [42].

#### 6.2.2. Diagnostic Accuracy and Meta-Analysis Results

The robustness of VI-RADS as a biomarker has been supported by extensive literature. Recent meta-analyses involving thousands of patients have consistently demonstrated a pooled sensitivity of 87–92% and a specificity of 85–88% for distinguishing NMIBC from MIBC [41,44]. Del Giudice et al. prospectively validated VI-RADS as a reliable tool to select high-risk NMIBC patients who may safely avoid repeat TURBT [45]. These high diagnostic performances support the increasing integration of VI-RADS into clinical practice. Nevertheless, VI-RADS scoring is not without limitations. Notably, the presence of a visible tumor stalk, which is typically associated with non-muscle-invasive disease, does not entirely exclude muscle invasion. Kim et al. reported a positive predictive value of 89% for the stalk sign on T2WI, indicating that approximately 11% of tumors with a visible stalk were ultimately confirmed as muscle-invasive on pathology [46], underscoring the inherent constraints of morphological assessment.

Table 2 summarizes the VI-RADS scoring criteria, including the characteristic MRI findings on T2WI, DWI, and DCE sequences for each score category, along with the corresponding probability of muscle invasion and recommended clinical management.

Although the accumulating evidence supports the growing clinical utility of mpMRI and VI-RADS, several limitations should be acknowledged. Most currently available studies are retrospective and heterogeneous in design, and large-scale prospective multicenter validation remains relatively limited. Accordingly, while mpMRI and VI-RADS are increasingly incorporated into clinical practice and international recommendations for local staging of bladder cancer, several applications—including treatment response assessment, prognostic stratification, and radiomics-based approaches—remain investigational and require further prospective validation before widespread routine adoption.

### 6.3. Technical Standardization: The Key to Biomarker Reliability

For an imaging biomarker to be clinically viable, it must demonstrate robust reproducibility across different institutions and equipment. Therefore, the standardization of MRI protocols is the cornerstone of the VI-RADS framework. However, achieving global standardization remains challenging owing to disparities in imaging resources and technology.

#### 6.3.1. 1.5 T vs. 3.0 T: Global Accessibility and Limitations

The universal adoption of mpMRI faces practical hurdles related to magnetic field strength. Generally, 3.0 Tesla (T) systems are preferred for bladder imaging owing to their superior signal-to-noise ratio (SNR) and increased spatial resolution, which are vital for identifying subtle disruptions within the thin layers of the bladder wall. In clinical practice, 1.5 T scanners remain the most widely utilized systems globally, particularly in community-based hospitals. Although 1.5-T systems are acceptable for VI-RADS assessment, they are inherently limited by lower SNR, often necessitating longer acquisition times or reduced spatial resolution. This technological gap underscores the importance of optimized protocols tailored to 1.5-T systems to ensure biomarker reliability regardless of local healthcare infrastructure [6,17].

#### 6.3.2. Sequence-Specific Slice Thickness Requirements

To minimize partial volume effects, i.e., where signals from different tissues are averaged within a single voxel, strict adherence to slice thickness recommendations is essential. The current consensus for maintaining biomarker integrity includes the following:

• T2WI: As the primary sequence for morphological staging, T2WI typically requires a slice thickness of approximately 3–4 mm. Thicker slices (e.g., ≥4–5 mm) increase the risk of blurring the interface between the tumor base and the muscularis propria, potentially leading to overstaging [6,17]. Beyond slice thickness, a small field of view (FOV) of approximately 20–24 cm and a high matrix size (e.g., 256 × 256 or higher) are recommended to maximize in-plane spatial resolution, which is essential for delineating the tumor–detrusor interface [6,17]. Furthermore, acquisition in at least two orthogonal planes (typically axial and either sagittal or coronal) is recommended to improve anatomical delineation and staging accuracy.
• DWI: To balance SNR and anatomical detail, a slice thickness of 3–4 mm is generally recommended. Ideally, DWI should closely match the T2WI slice thickness to allow accurate side-by-side comparison during VI-RADS scoring. The selection of appropriate b-values is paramount; a minimum of two b-values is required, with a high b-value of ≥800 s/mm^2^ recommended to maximize diffusion contrast and improve the conspicuity of restricted diffusion within tumor tissue [6,17]. Although axial imaging is fundamental, the inclusion of an additional plane, such as coronal DWI, may further improve lesion localization and diagnostic confidence.
• DCE Imaging: High spatial resolution is indispensable for DCE imaging, and a thinner slice thickness of approximately 1–2 mm is recommended to accurately capture the early submillimetric enhancement of the inner bladder wall, which is a key indicator of early muscle invasion. Along with spatial resolution, adequate temporal resolution—ideally with a scan acquisition time of <10 s per phase—is necessary to reliably capture the early arterial enhancement phase, which distinguishes tumor tissue from the surrounding bladder wall [6,17].

Adherence to these sequence-specific parameters, in conjunction with appropriate patient preparation, forms the technical foundation for reliable and reproducible VI-RADS scoring across different institutions and scanner platforms.

#### 6.3.3. Patient Preparation and Artifact Mitigation

The reliability of an imaging biomarker is influenced by the patient’s physiological condition during image acquisition. Adequate bladder distension is critical for accurate evaluation: an underdistended bladder may mimic wall thickening, whereas overdistension can obscure subtle tumor invasion and introduce motion-related artifacts. Ideally, an appropriate bladder distension of 300–500 mL should be achieved for optimal visualization [6,17]. To ensure this volume consistently, a standardized hydration protocol is recommended: patients should void urine 2 h before the scan and drink 500–1000 mL of water 30 min before the acquisition. This controlled filling prevents the collapsed state that often leads to diagnostic uncertainty. Furthermore, the use of antiperistaltic agents (e.g., hyoscine butylbromide) is recommended to reduce bowel-related motion artifacts, particularly on DWI, which may otherwise compromise the reliability of ADC measurements [5,6,17].

Beyond patient preparation immediately prior to scanning, the timing of mpMRI relative to prior interventions is equally important. When mpMRI is performed after TURBT, bladder biopsy, or intravesical therapy including BCG or chemotherapy instillation, edema and inflammation can develop within the bladder wall and perivesical tissue and lead to significant alterations in imaging characteristics [5,6]. To minimize these diagnostic pitfalls, mpMRI should be scheduled at least 2 weeks after such procedures; a 2–3 day interval after cystoscopy or Foley catheter removal is recommended to avoid DWI susceptibility artifacts caused by residual air in the bladder [5,8]. Notably, these recommendations are based on expert consensus rather than definitive scientific evidence [5,6]. Therefore, adherence to these protocols is important to ensure the reproducibility and reliability of VI-RADS-based assessments.

### 6.4. Emergence of Biparametric MRI (bpMRI)

As the field moves toward value-based healthcare, the necessity of the DCE sequence has been increasingly questioned. bpMRI, consisting of T2WI and DWI, has been proposed as a faster, more cost-effective, and safer alternative by eliminating the need for gadolinium-based contrast agents. This approach reduces scan time and avoids potential risks associated with contrast administration, including nephrogenic systemic fibrosis in patients with impaired renal function, as well as emerging concerns regarding gadolinium deposition in the brain and its potential association with parkinsonism [47,48,49].

Recent studies have demonstrated that bpMRI can achieve diagnostic performance comparable to mpMRI for predicting muscle invasion in bladder cancer, with similar sensitivity and specificity reported across multiple cohorts [50,51,52]. Notably, DWI plays a central role as a functional biomarker, enabling the assessment of tumor cellularity and improving detection of tumor infiltration, whereas T2WI provides essential anatomical delineation of the bladder wall.

However, the omission of DCE imaging remains controversial. DCE offers complementary information on tumor vascularity and early enhancement patterns, which may improve diagnostic confidence in challenging cases. DCE is of particular value when DWI is degraded by susceptibility artifacts or when differentiating tumor tissue from post-treatment changes such as fibrosis or inflammation.

From a clinical perspective, bpMRI may be suitable for initial risk stratification, surveillance, and use in resource-limited settings. In contrast, mpMRI remains preferable in equivocal cases or when precise local staging is critical for treatment planning. Therefore, bpMRI and mpMRI should be regarded as complementary approaches rather than as competing approaches, reflecting the evolving role of imaging biomarkers in balancing diagnostic accuracy, efficiency, and clinical applicability.

## 7. Future Perspectives: Radiomics, Artificial Intelligence, and Treatment Monitoring

Although the mpMRI and VI-RADS framework has significantly improved the standardization of bladder cancer staging, the field is rapidly evolving toward a more personalized and computational approach. The integration of radiomics and AI is transforming imaging from a visual interpretation tool into a high-dimensional data source. Furthermore, the clinical utility of mpMRI is expanding beyond its application in initial diagnosis to the critical role of monitoring and predicting response to systemic therapies.

### 7.1. Radiomics: Extracting the “Invisible” Biomarkers

Radiomics involves the high-throughput extraction of quantitative features, which are often imperceptible to the human eye, from medical images. These include tumor shape, signal intensity distribution, and texture patterns (e.g., entropy, homogeneity) [53]. In the context of bladder cancer, radiomics serves as a powerful imaging biomarker for two key clinical challenges. First, regarding the distinction of MIBC from NMIBC, several studies have demonstrated that radiomic features extracted from T2WI and DWI can achieve higher diagnostic accuracy than traditional VI-RADS scoring alone, particularly in the equivocal VI-RADS 3 category. By analyzing the heterogeneity of the tumor–bladder wall interface, radiomic models can identify microscopic muscle invasion with high sensitivity [54,55]. Second, beyond staging, radiomics has shown potential in predicting tumor grade (low vs. high grade) and even specific molecular subtypes. This “virtual biopsy” capability may be invaluable for patients who cannot undergo aggressive TURBT due to comorbidities [55,56].

### 7.2. AI and Deep Learning (DL): Towards Automated Precision

The application of AI, particularly DL and convolutional neural networks (CNNs), has shown significant potential in addressing key limitations of human interpretation, including inter-observer variability and the time-consuming nature of manual image analysis. Recent DL-based approaches, including general CNN architectures such as HyperDense-Net [57], have been applied to automated bladder-tumor segmentation on mpMRI, with comparative studies demonstrating improved performance across different architectures [58]. AI-driven models have also been explored for automated risk stratification and staging, with emerging studies demonstrating promising performance in tumor detection and classification tasks, potentially facilitating the broader adoption of mpMRI in non-academic and community settings where subspecialty expertise may be limited [58,59].

Despite these advances, several challenges remain. A major limitation is the black box nature of many AI systems, where the internal decision-making process is not readily interpretable, limiting clinical trust and hindering integration into routine practice. Accordingly, explainable artificial intelligence (XAI) approaches have gained increasing attention as a strategy to improve transparency, interpretability, and clinician trust in AI-assisted decision-making. XAI frameworks may also play an important role in regulatory approval and clinical implementation by enabling clinicians to better understand the rationale underlying AI-generated predictions and classifications. Increasing attention has therefore been directed toward multimodal large language models (LLMs), which integrate imaging data with clinical and textual information to generate more context-aware outputs and have the potential to enhance decision support and improve communication between AI systems and clinicians [60,61,62]. Further validation through large-scale, multi-center studies and the development of standardized frameworks will be essential for clinical translation.

### 7.3. Monitoring and Predicting Response to Systemic Therapy

A transformative application of mpMRI as an imaging biomarker is its expanding role in monitoring response to systemic treatments, such as NAC and immunotherapy. This shift from static staging to dynamic response assessment is critical for personalized medicine in MIBC.

#### 7.3.1. Neoadjuvant Immunotherapy Assessment (The PURE-01 Study)

The PURE-01 study was among the first to demonstrate the potential of neoadjuvant pembrolizumab in patients with MIBC, reporting high pathological complete response (pCR) rates and establishing proof-of-concept for immune checkpoint inhibition in the preoperative setting [63]. Importantly, this study explored the role of mpMRI as a non-invasive imaging biomarker for treatment-response assessment. In an exploratory analysis, changes in tumor morphology, diffusion restriction, and contrast enhancement patterns on mpMRI were preliminarily associated with pathological response, suggesting that mpMRI enables the early prediction of treatment efficacy prior to radical cystectomy [63]. Initial analyses further indicated that imaging findings could complement molecular biomarkers such as PD-L1 expression and tumor mutational burden, supporting a multimodal approach to response assessment. Subsequent follow-up studies have confirmed the durability of these findings, with sustained survival benefits observed at both 3-year and 5-year timepoints, reinforcing the prognostic significance of pathological response to neoadjuvant pembrolizumab [64,65]. Collectively, these clinical outcomes provide an important framework supporting the utility of mpMRI-based response assessment in the context of neoadjuvant immunotherapy.

#### 7.3.2. Standardizing Response with VI-RADS

To ensure reproducibility in treatment monitoring, the VI-RADS framework is being adapted for post-systemic therapy settings. Pecoraro et al. proposed the NAC VI-RADS (nacVI-RADS) scoring system to evaluate the response to NAC in patients with MIBC who had previously undergone TURBT [43]. In this clinically relevant setting, wherein maximal TURBT is typically performed prior to systemic therapy to reduce tumor burden, nacVI-RADS aims to differentiate complete from partial radiological response (Figure 4). However, the interpretation of post-treatment imaging may be confounded by combined effects of prior resection and chemotherapy.

#### 7.3.3. Clinical Decision-Making Support

The integration of multiple MRI sequences (T2WI, DWI, and DCE) is essential for comprehensive response evaluation. Bandini et al. have emphasized the complementary role of these mpMRI sequences in supporting clinical decision-making in MIBC, particularly when determining whether to proceed with radical cystectomy or consider bladder-sparing strategies in selected responders [66].

#### 7.3.4. Implications for Bladder Preservation

By providing a non-invasive assessment of residual tumor burden, mpMRI may assist in identifying patients who have achieved a favorable response to systemic therapy. This approach has the potential to support the selection of appropriate candidates for bladder-sparing strategies, although further prospective validation is required before routine clinical implementation.

### 7.4. The “Radiogenomics” Frontier: Integrated Diagnostics

The ultimate goal of non-invasive biomarkers is the integration of imaging and molecular data, which is collectively known as radiogenomics. By correlating mpMRI phenotypes with genomic alterations such as *TERT* promoter mutations and *FGFR3* status, radiogenomics aims to provide an integrated, patient-specific biomarker profile [67].

Radiogenomics extends conventional radiomics by integrating quantitative imaging features with genomic and transcriptomic data, enabling more thorough tumor phenotype characterization and outcome prediction. In bladder cancer, radiogenomic nomograms have demonstrated encouraging performance, with median AUC values of 0.83 in training sets and 0.75 in validation sets. Emerging evidence further suggests that tumors with distinct molecular characteristics exhibit specific imaging phenotypes, including variations in diffusion restriction and enhancement patterns [67]. In particular, an MRI/RNA-seq-based radiogenomics model integrating imaging with transcriptomic data achieved 94% sensitivity, 88% specificity, and 92% accuracy in differentiating intra-bladder from extra-bladder disease, outperforming models based on genetic or radiomic data alone [68]. Additionally, molecular subtype classification into luminal and basal subtypes has been shown to be recognizable through MRI radiomic features, enabling non-invasive immune prognostic profiling [69].

The integration of imaging biomarkers with liquid-biopsy approaches, including ctDNA and urine-based genomic assays, may further enhance risk stratification and treatment personalization [13,14,15,16,32,33]. Although large-scale prospective validation remains essential, radiogenomics represents a promising frontier that moves beyond anatomical staging towards a biologically informed and patient-specific characterization of disease and is expected to play an increasingly central role in guiding individualized treatment decisions in bladder cancer.

Despite its potential, several logistical and technological hurdles currently limit the routine integration of radiogenomics into multidisciplinary team meetings. Logistically, the labor-intensive nature of manual tumor segmentation and feature extraction may create a significant bottleneck, making it difficult to provide results within the rapid turnaround time required for clinical decision-making. Furthermore, limited interoperability between radiology PACS systems and genomic databases hinders seamless data integration. Technologically, variability in imaging protocols across institutions presents challenges for model reproducibility and generalizability. Therefore, broader clinical integration of radiogenomics will likely require more automated and standardized analytical pipelines capable of providing interpretable and clinically applicable results in a timely manner.

## 8. Conclusions

Bladder cancer remains a highly heterogeneous disease characterized by variable clinical behavior and a high risk of recurrence, underscoring the need for accurate and reliable risk stratification. Although cystoscopy and histopathological evaluation continue to serve as the reference standards, they are limited by invasiveness, sampling errors, and interobserver variability.

In this context, non-invasive biomarkers, including urine-based, blood-based, and imaging biomarkers, have emerged as promising tools to complement conventional diagnostic approaches. Urine-based molecular assays and blood-based liquid-biopsy approaches are promising adjunctive tools for risk stratification and surveillance; however, their clinical adoption remains limited by issues of standardization and cost. Among imaging biomarkers, mpMRI, particularly within the framework of VI-RADS, has demonstrated encouraging diagnostic performance and growing clinical utility in local staging and prognostic assessment.

Recent advances in imaging biomarkers, including DWI, radiomics, and AI-based analysis, have further enhanced the ability of mpMRI to characterize tumor biology and predict treatment response. In parallel, the integration of imaging with molecular biomarkers through radiogenomics and multimodal analytical approaches represents an important step toward precision oncology.

Despite these advances, several challenges remain. Variability in imaging protocols, lack of standardization across institutions, and limited prospective validation continue to restrict widespread clinical implementation. Moreover, no single non-invasive biomarker has demonstrated sufficient accuracy to replace cystoscopy in routine practice. In addition, practical implementation remains influenced by healthcare system resources, cost-effectiveness considerations, imaging accessibility, and the availability of specialized expertise, which may vary substantially across institutions and geographic regions.

Future research should focus on large-scale, multi-center validation studies, the standardization of imaging protocols, and the development of integrated multimodal frameworks combining imaging, molecular, and clinical data. Such efforts will be critical for the translation of these advances into clinical practice, ultimately enabling a shift from anatomical staging alone towards a more integrated and biology-informed approach to bladder cancer management.

## Figures and Tables

**Table 1:** Comparison of non-invasive urinary, blood-based, and imaging-based biomarkers in bladder cancer.

Category	Biomarker Type	Examples	Clinical Utility	Advantages	Limitations	Level of Evidence	Cost	Clinical Readiness	Validation Source
Urine-based	Protein-based	NMP22, BTA-stat, BTA-TRAK	Adjunct to cytology; screening and surveillance	Higher sensitivity than cytology; FDA-approved	Low specificity; false positives in UTI, hematuria, and intravesical inflammation	Meta-analysis/prospective multicenter	Low	Current	Mixed: FDA clearance trials by Matritech (NMP22) and Polymedco/Bion (BTA) were industry-funded; subsequent comparative studies are largely academic and independent
	Genomic/molecular	FGFR3, TERT promoter, PIK3CA mutations; DNA methylation panels (e.g., Bladder EpiCheck)	Detection of early-stage tumors; NMIBC surveillance	High diagnostic accuracy; may detect molecular alteration before visible cystoscopic recurrence	High cost; specialized infrastructure; lack of standardized reporting	Prospective single-center/retrospective	High	Current (limited availability)	Mixed: FGFR3/TERT mutation studies are academic; Bladder EpiCheck pivotal trial was sponsored by Nucleix with sponsor involvement in design and analysis
	Liquid biopsy (utDNA)	Urinary tumor DNA (utDNA); cell-free DNA via ddPCR or NGS	MRD detection after radical cystectomy; NMIBC surveillance; may precede radiologic or cystoscopic findings	Detection of minute DNA quantities; high NPV; non-invasive serial monitoring	Influenced by the molecular field effect and pre-analytical variables (e.g., urine concentration, sample timing)	Prospective single-center/retrospective	High	Future (investigational)	Independent: investigator-initiated studies at academic medical centers; no single commercial sponsor or proprietary kit dependency
Blood-based	ctDNA	Plasma cell-free tumor DNA (cf-DNA)	MRD detection post-cystectomy; monitoring NAC and adjuvant immunotherapy response	Precedes radiologic progression; systemic tumor burden monitoring	Low sensitivity in early-stage NMIBC; high sequencing cost	Prospective multicenter	High	Current (MIBC/advanced disease)	Mixed: IMvigor series (Roche/Genentech-sponsored); TOMBOLA and MODERN trials are publicly funded by academic consortia
	CTCs	Intact circulating tumor cells	Prognostication in MIBC; assessment of systemic dissemination	Reflects metastatic potential; potential for PD-L1 profiling and immunotherapy selection	Technical challenges in isolation; low CTC count in localized disease	Retrospective/prospective single-center	High	Future (investigational)	Mixed: CellSearch platform (Menarini) required for isolation; key trials (e.g., CirGuidance) were investigator-initiated independent studies
	Systemic inflammatory markers	NLR (neutrophil-to-lymphocyte ratio)	Baseline risk stratification; prognostic indicator in NMIBC and MIBC	Cost-effective; readily available; easily integrated into routine clinical practice	Lack of tumor specificity; influenced by systemic inflammation, infection, or steroid use	Meta-analysis/retrospective	Low	Current	Independent: all published meta-analyses are from academic institutions; no commercial sponsor; no proprietary assay involved
Imaging-based	mpMRI (multiparametric MRI)	T2WI + DWI + DCE; VI-RADS scoring system	Local staging (NMIBC vs. MIBC); treatment response monitoring (NAC, immunotherapy); risk stratification prior to TURBT or cystectomy	Favorable diagnostic performance in meta-analyses: standardized VI-RADS framework; functional and morphological assessment; no radiation exposure	Requires gadolinium contrast; higher cost and scan time; limited availability in community settings; susceptibility to post-TURBT artifacts; requires expertise for interpretation	Meta-analysis/prospective multicenter	Moderate	Current (specialized centers)	Independent: VI-RADS developed by academic radiology consortium; multicenter validation non-commercial; endorsed by EAU 2024 guidelines
	bpMRI (bi-parametric MRI)	T2WI + DWI only (no contrast); simplified VI-RADS assessment	Initial risk stratification; surveillance in resource-limited settings; suitable for patients with contraindications to gadolinium	No gadolinium required (avoids nephrogenic systemic fibrosis risk and gadolinium deposition); shorter scan time; relatively lower cost; comparable diagnostic performance to mpMRI in selected cohorts	Omission of DCE limits assessment of tumor vascularity; reduced diagnostic confidence in equivocal cases or post-treatment settings; DWI susceptible to motion and susceptibility artifacts	Prospective single-center/retrospective	Moderate	Current (specialized centers)	Independent: academic single/multicenter prospective studies and institutional registries; no commercial sponsorship concern

BTA, bladder tumor antigen; cfDNA, cell-free DNA; ctDNA, circulating tumor DNA; CTCs, circulating tumor cells; ddPCR, digital droplet polymerase chain reaction; FDA, US Food and Drug Administration; FGFR3, fibroblast growth factor receptor 3; MIBC, muscle-invasive bladder cancer; MRD, minimal residual disease; NAC, neoadjuvant chemotherapy; NGS, next-generation sequencing; NLR, neutrophil-to-lymphocyte ratio; NMIBC, non-muscle-invasive bladder cancer; NMP22, nuclear matrix protein 22; NPV, negative predictive value; PD-L1, programmed death-ligand 1; PIK3CA, phosphatidylinositol-4,5-bisphosphate 3-kinase catalytic subunit alpha; TERT, telomerase reverse transcriptase; UTI, urinary tract infection; utDNA, urinary tumor DNA; VI-RADS, Vesical Imaging-Reporting and Data System. Level of Evidence: Meta-analysis/Prospective multicenter = highest; Prospective single-center = moderate; Retrospective = lower. Validation Source: Independent = no commercial sponsor or proprietary assay involvement; Mixed = combination of industry-sponsored pivotal data and independent academic validation; Industry-sponsored pivotal data should be interpreted with awareness of potential sponsor bias. Cost: Low = routine clinical use; Moderate = institutional availability with standard equipment; High = specialized infrastructure required. Clinical Readiness: Current = clinically available; Current (limited availability) = available but institution-dependent; Future = investigational. Cost stratification is relative and based on required infrastructure and reagent costs: Low = standard laboratory; Moderate = institutional imaging equipment; High = specialized sequencing platform or proprietary assay kit.

**Table 2:** VI-RADS scoring criteria: MRI findings and clinical implications.

VI-RADS	T2WI	DWI/ADC	DCE	Muscle Invasion	Clinical Implication
1	Small papillary tumor (≤1 cm) with or without visible stalk; intact low-signal intensity (SI) muscularis propria	Restricted diffusion confined to stalk; ADC reduction in tumor only	Early mucosal enhancement without inner-layer thickening or muscularis-propria disruption	Very unlikely	TURBT as primary treatment; low-risk surveillance
2	Papillary tumor (>1 cm) with thickened inner layer and tumor stalk sign; intact muscularis propria	Inchworm sign: diffusion restriction confined to tumor stalk; no extension into muscle	Early enhancement of inner layer; muscularis propria not involved	Unlikely	TURBT ± intravesical therapy; standard NMIBC management
3	Disappearance of category 2 findings, but no clear disruption of low SI muscularis propria	Loss or indistinct inchworm sign; diffusion restriction with equivocal muscular involvement	Equivocal early enhancement; inner-layer involvement uncertain	Equivocal	Re-TURBT or closer surveillance; consider mpMRI-guided decision
4	Sessile tumor with the disruption of low SI muscularis propria; no extravesical extension	Marked restriction extending into muscularis propria	Early transmural enhancement extending through muscularis propria	Likely	Neoadjuvant chemotherapy + radical cystectomy; CCRT in selected patients
5	Direct tumor extension into perivesical fat or adjacent organs	Diffuse marked restriction beyond bladder wall	Avid heterogeneous enhancement with extravesical extension	Very high/definitive	Radical cystectomy or multimodal therapy; staging workup for metastasis

ADC, apparent diffusion coefficient; CCRT, concurrent chemoradiotherapy; DCE, dynamic contrast-enhanced; DWI, diffusion-weighted imaging; mpMRI, multiparametric MRI; MRI, magnetic resonance imaging; NMIBC, non-muscle-invasive bladder cancer; T2WI, T2-weighted imaging; TURBT, transurethral resection of bladder tumor; VI-RADS, Vesical Imaging-Reporting and Data System. Adapted from: [5,17].

## Data Availability

No new data were created or analyzed in this study.

## Abbreviations

The following abbreviations are used in this manuscript:

ADC	apparent diffusion coefficient
AI	artificial intelligence
AUC	area under the curve
BCG	Bacillus Calmette-Guérin
bpMRI	biparametric MRI
BTA	bladder Tumor Antigen
CCRT	concurrent chemoradiotherapy
cfDNA	cell-free DNA
CIS	carcinoma in situ
CNNs	convolutional neural networks
CT	computed tomography
CTCs	circulating tumor cells
ctDNA	circulating tumor DNA
DCE	dynamic contrast-enhanced
ddPCR	digital droplet polymerase chain reaction
DL	deep learning
DWI	diffusion-weighted imaging
FDA	US Food and Drug Administration
LLMs	large language models
MIBC	muscle-invasive bladder cancer
mpMRI	multiparametric magnetic resonance imaging
MRI	magnetic resonance imaging
NAC	neoadjuvant chemotherapy
nacVI-RADS	NAC VI-RADS
NGS	next-generation sequencing
NLR	neutrophil-to-lymphocyte ratio
NMIBC	non–muscle-invasive bladder cancer
NMP22	nuclear matrix protein 22
pCR	pathological complete response
SNR	signal-to-noise ratio
T2WI	T2-Weighted Imaging
TURBT	transurethral resection of bladder tumor
utDNA	urinary tumor DNA
VI-RADS	Vesical Imaging Reporting and Data System

## References

[1] Bray F., Laversanne M., Sung H., Ferlay J., Siegel R.L., Soerjomataram I., Jemal A. (2024). Global cancer statistics 2022: GLOBOCAN estimates of incidence and mortality worldwide for 36 cancers in 185 countries. CA Cancer J. Clin..

[2] Holzbeierlein J.M., Bixler B.R., Buckley D.I., Chang S.S., Holmes R., James A.C., Kirkby E., McKiernan J.M., Schuckman A.K. (2024). Diagnosis and treatment of non-muscle invasive bladder cancer: AUA/SUO guideline: 2024 amendment. J. Urol..

[3] Gontero P., Birtle A., Capoun O., Compérat E., Dominguez-Escrig J.L., Liedberg F., Mariappan P., Masson-Lecomte A., Mostafid H.A., Pradere B. (2024). European Association of Urology guidelines on non-muscle-invasive bladder cancer (TaT1 and carcinoma in situ)—A summary of the 2024 guidelines update. Eur. Urol..

[4] Zhang Y., Rumgay H., Li M., Yu H., Pan H., Ni J. (2023). The global landscape of bladder cancer incidence and mortality in 2020 and projections to 2040. J. Glob. Health.

[5] Pecoraro M., Cipollari S., Messina E., Laschena L., Dehghanpour A., Borrelli A., Del Giudice F., Muglia V.F., Vargas H.A., Panebianco V. (2025). Multiparametric MRI for bladder cancer: A practical approach to the clinical application of VI-RADS. Radiology.

[6] Fávero Prietto Dos Santos J., Ghezzi C.L.A., Pedrollo I.M., Cruz Í.R., Orozco O.F.G., Zapparoli M., Schuch A., Muglia V.F. (2024). Practical guide to VI-RADS: MRI protocols, lesion characterization, and pitfalls. Radiographics.

[7] Magers M.J., Lopez-Beltran A., Montironi R., Williamson S.R., Kaimakliotis H.Z., Cheng L. (2019). Staging of bladder cancer. Histopathology.

[8] Sim K.C., Sung D.J. (2020). Role of magnetic resonance imaging in tumor staging and follow-up for bladder cancer. Transl. Androl. Urol..

[9] Richterstetter M., Wullich B., Amann K., Haeberle L., Engehausen D.G., Goebell P.J., Krause F.S. (2012). The value of extended transurethral resection of bladder tumour (TURBT) in the treatment of bladder cancer. BJU Int..

[10] Ark J.T., Keegan K.A., Barocas D.A., Morgan T.M., Resnick M.J., You C., Cookson M.S., Penson D.F., Davis R., Clark P.E. (2014). Incidence and predictors of understaging in patients with clinical T1 urothelial carcinoma undergoing radical cystectomy. BJU Int..

[11] Cumberbatch M.G.K., Foerster B., Catto J.W.F., Kamat A.M., Kassouf W., Jubber I., Shariat S.F., Sylvester R.J., Gontero P. (2018). Repeat transurethral resection in non-muscle-invasive bladder cancer: A systematic review. Eur. Urol..

[12] Gontero P., Sylvester R., Pisano F., Joniau S., Oderda M., Serretta V., Larré S., Di Stasi S., Van Rhijn B., Witjes A.J. (2016). The impact of re-transurethral resection on clinical outcomes in a large multicentre cohort of patients with T1 high-grade/Grade 3 bladder cancer treated with bacille Calmette-Guérin. BJU Int..

[13] Jain M., Tivtikyan A., Kislyakov D., Rakhmatullin T., Kamalov D., Kokarev V., Vorobeva L., Samokhodskaya L., Zvereva M., Kamalov A. (2025). The diagnostic performance of a four-gene digital droplet PCR panel for urine liquid biopsy in urothelial bladder cancer. Diagnostics.

[14] Rodas Garzaro J.R., Kravchuk A., Burger M., Wolff I., Lebentrau S., Rubio-Briones J., Brás J.P., Gilfrich C., Siepmann S., Pahernik S. (2026). Diagnostic performance and clinical utility of the Uromonitor^®^ molecular urine assay for urothelial carcinoma of the bladder: A systematic review and diagnostic accuracy meta-analysis. Diagnostics.

[15] Song Y., Jiang S., Peng Y., Du Y., Qin C., Xu T. (2025). FGFR3 alteration sites and response rate to FGFR inhibitors in urothelial carcinoma. Pharmacol. Res..

[16] Song Y., Du Y., Jiang S., Peng Y., Luo X., Xu T. (2025). Efficacy and safety of selective pan-fibroblast growth factor receptor (FGFR) tyrosine kinase inhibitors in FGFR-altered urothelial carcinoma. Pharmacol. Res..

[17] Panebianco V., Narumi Y., Altun E., Bochner B.H., Efstathiou J.A., Hafeez S., Huddart R., Kennish S., Lerner S., Montironi R. (2018). Multiparametric magnetic resonance imaging for bladder cancer: Development of VI-RADS (Vesical Imaging-Reporting and Data System). Eur. Urol..

[18] Rosser C.J., Urquidi V., Goodison S. (2013). Urinary biomarkers of bladder cancer: An update and future perspectives. Biomark. Med..

[19] Chen F., Lin X. (2023). The Paris system for reporting urinary cytology: An updated review. J. Clin. Transl. Pathol..

[20] Barkan G.A., Wojcik E.M., Nayar R., Savic-Prince S., Quek M.L., Kurtycz D.F.I., Rosenthal D.L. (2016). The Paris system for reporting urinary cytology: The quest to develop a standardized terminology. Acta Cytol..

[21] Wojcik E.M., Kurtycz D.F.I., Rosenthal D.L. (2022). We’ll always have Paris. The Paris System for Reporting Urinary Cytology 2022. J. Am. Soc. Cytopathol..

[22] Michałek I.M., Durzyńska M., dos Santos F.L.C. (2024). The Paris System for Reporting Urinary Cytology—A critical review of its role in advancing precision diagnostics with insights into artificial intelligence integration. Nowotw. J. Oncol..

[23] Collado A., Chéchile G.E., Salvador J., Vicente J. (2000). Early complications of endoscopic treatment for superficial bladder tumors. J. Urol..

[24] Van Der Meijden A., Sylvester R., Collette L., Bono A., Ten Kate F. (2000). The role and impact of pathology review on stage and grade assessment of stages Ta and T1 bladder tumors: A combined analysis of 5 European Organization for Research and Treatment of Cancer Trials. J. Urol..

[25] Chou R., Gore J.L., Buckley D., Fu R., Gustafson K., Griffin J.C., Grusing S., Selph S. (2015). Urinary biomarkers for diagnosis of bladder cancer: A systematic review and meta-analysis. Ann. Intern. Med..

[26] Lee H.-H., Kim S.H. (2020). Review of non-invasive urinary biomarkers in bladder cancer. Transl. Cancer Res..

[27] Tomiyama E., Fujita K., Hashimoto M., Uemura H., Nonomura N. (2024). Urinary markers for bladder cancer diagnosis: A review of current status and future challenges. Int. J. Urol..

[28] Witjes J.A., Morote J., Cornel E.B., Gakis G., van Valenberg F.J.P., Lozano F., Sternberg I.A., Willemsen E., Hegemann M.L., Paitan Y. (2018). Performance of the Bladder EpiCheck™ methylation test for patients under surveillance for non-muscle-invasive bladder cancer: Results of a multicenter, prospective, blinded clinical trial. Eur. Urol. Oncol..

[29] Chauhan P.S., Chen K., Babbra R.K., Feng W., Pejovic N., Nallicheri A., Harris P.K., Dienstbach K., Atkocius A., Maguire L. (2021). Urine tumor DNA detection of minimal residual disease in muscle-invasive bladder cancer treated with curative-intent radical cystectomy: A cohort study. PLoS Med..

[30] Majewski T., Yao H., Bondaruk J., Chung W., Lee S., Lee J.G., Zhang S., Cogdell D., Yang G., Choi W. (2019). Whole-organ genomic characterization of mucosal field effects initiating bladder carcinogenesis. Cell Rep..

[31] Knowles M.A., Hurst C.D. (2015). Molecular biology of bladder cancer: New insights into pathogenesis and clinical diversity. Nat. Rev. Cancer.

[32] Christensen E., Birkenkamp-Demtröder K., Sethi H., Shchegrova S., Salari R., Nordentoft I., Wu H.T., Knudsen M., Lamy P., Lindskrog S.V. (2019). Early detection of metastatic relapse and monitoring of therapeutic efficacy by ultra-deep sequencing of plasma cell-free DNA in patients with urothelial bladder carcinoma. J. Clin. Oncol..

[33] Powles T., Assaf Z.J., Davarpanah N., Banchereau R., Szabados B.E., Yuen K.C., Grivas P., Hussain M., Oudard S., Gschwend J.E. (2021). ctDNA guiding adjuvant immunotherapy in urothelial carcinoma. Nature.

[34] Zulfiqqar A., Liliana B., Mataho N.L., Subekti E. (2025). The use of circulating tumor cells in T1 stage non-muscle invasive bladder cancer: A systematic review and meta-analysis. Urol. Res. Pract..

[35] Vartolomei M.D., Porav-Hodade D., Ferro M., Mathieu R., Abufaraj M., Foerster B., Kimura S., Shariat S.F. (2018). Prognostic role of pretreatment neutrophil-to-lymphocyte ratio (NLR) in patients with non-muscle-invasive bladder cancer (NMIBC): A systematic review and meta-analysis. Urol. Oncol..

[36] Mano R., Baniel J., Shoshany O., Margel D., Bar-On T., Nativ O., Rubinstein J., Halachmi S. (2015). Neutrophil-to-lymphocyte ratio predicts progression and recurrence of non-muscle-invasive bladder cancer. Urol. Oncol..

[37] Panebianco V., Pecoraro M., Del Giudice F., Takeuchi M., Muglia V.F., Messina E., Cipollari S., Giannarini G., Catalano C., Narumi Y. (2022). VI-RADS for bladder cancer: Current applications and future developments. J. Magn. Reson. Imaging.

[38] Takeuchi M., Sasaki S., Ito M., Okada S., Takahashi S., Kawai T., Suzuki K., Oshima H., Hara M., Shibamoto Y. (2009). Urinary bladder cancer: Diffusion-weighted MR imaging—Accuracy for diagnosing T stage and estimating histologic grade. Radiology.

[39] Kobayashi S., Koga F., Kajino K., Yoshita S., Ishii C., Tanaka H., Saito K., Masuda H., Fujii Y., Yamada T. (2014). Apparent diffusion coefficient value reflects invasive and proliferative potential of bladder cancer. J. Magn. Reson. Imaging.

[40] Barchetti G., Simone G., Ceravolo I., Salvo V., Campa R., Del Giudice F., De Berardinis E., Buccilli D., Catalano C., Gallucci M. (2019). Multiparametric MRI of the bladder: Inter-observer agreement and accuracy with the Vesical Imaging-Reporting and Data System (VI-RADS) at a single reference center. Eur. Radiol..

[41] Woo S., Panebianco V., Narumi Y., Del Giudice F., Muglia V.F., Takeuchi M., Ghafoor S., Bochner B.H., Goh A.C., Hricak H. (2020). Diagnostic performance of vesical imaging reporting and data system for the prediction of muscle-invasive bladder cancer: A systematic review and meta-analysis. Eur. Urol. Oncol..

[42] Pecoraro M., Takeuchi M., Vargas H.A., Muglia V.F., Cipollari S., Catalano C., Panebianco V. (2020). Overview of VI-RADS in bladder cancer. AJR Am. J. Roentgenol..

[43] Pecoraro M., Del Giudice F., Magliocca F., Simone G., Flammia S., Leonardo C., Messina E., De Berardinis E., Cortesi E., Panebianco V. (2022). Vesical Imaging-Reporting and Data System (VI-RADS) for assessment of response to systemic therapy for bladder cancer: Preliminary report. Abdom. Radiol..

[44] Del Giudice F., Flammia R.S., Pecoraro M., Moschini M., D’Andrea D., Messina E., Pisciotti L.M., De Berardinis E., Sciarra A., Panebianco V. (2022). The accuracy of Vesical Imaging-Reporting and Data System (VI-RADS): An updated comprehensive multi-institutional, multi-readers systematic review and meta-analysis from diagnostic evidence into future clinical recommendations. World J. Urol..

[45] Del Giudice F., Barchetti G., De Berardinis E., Pecoraro M., Salvo V., Simone G., Sciarra A., Leonardo C., Gallucci M., Catalano C. (2020). Prospective assessment of Vesical Imaging Reporting and Data System (VI-RADS) and its clinical impact on the management of high-risk non-muscle-invasive bladder cancer patients candidate for repeated transurethral resection. Eur. Urol..

[46] Kim D.H., Kang B.C., Chung J. (2020). T1-staging for urinary bladder cancer with the stalk and inchworm signs with 3.0 Tesla MRI. J. Korean Soc. Radiol..

[47] Alabousi M., Davenport M.S. (2021). Use of intravenous gadolinium-based contrast media in patients with kidney disease and the risk of nephrogenic systemic fibrosis: Radiology in training. Radiology.

[48] Shin N.-Y., Park S.K., Kim B., Han K., Han K., Kim J., Lee S.-K., Ahn S.V. (2026). Risk of parkinsonism after exposure to different types of gadolinium-based contrast agents: A nationwide population-based cohort study of 222,977 individuals. Korean J. Radiol..

[49] Kim C., Kim C., Tae B.S., Kwon D.Y., Lee Y.H. (2025). Assessing the association between gadolinium-based contrast agents and Parkinson disease: Insights from the Korean National Health Insurance Service database. Investig. Radiol..

[50] Liu P., Cai L., Jiang L., Chen H., Cao Q., Bai K., Bai R., Wu Q., Yang X., Lu Q. (2025). Comparative diagnostic performance of VI-RADS based on biparametric and multiparametric MRI in predicting muscle invasion in bladder cancer. BMC Med. Imaging.

[51] Noh T.I., Shim J.S., Kang S.G., Sung D.J., Cheon J., Sim K.C., Kang S.H. (2022). Comparison between biparametric and multiparametric MRI in predicting muscle invasion by bladder cancer based on the VI-RADS. Sci. Rep..

[52] Watanabe M., Taguchi S., Machida H., Tambo M., Takeshita Y., Kariyasu T., Fukushima K., Shimizu Y., Okegawa T., Fukuhara H. (2022). Clinical validity of non-contrast-enhanced VI-RADS: Prospective study using 3-T MRI with high-gradient magnetic field. Eur. Radiol..

[53] Lambin P., Rios-Velazquez E., Leijenaar R., Carvalho S., van Stiphout R.G.P.M., Granton P., Zegers C.M.L., Gillies R., Boellard R., Dekker A. (2012). Radiomics: Extracting more information from medical images using advanced feature analysis. Eur. J. Cancer.

[54] Boca B., Caraiani C., Telecan T., Pintican R., Lebovici A., Andras I., Crisan N., Pavel A., Diosan L., Balint Z. (2023). MRI-based radiomics in bladder cancer: A systematic review and radiomics quality score assessment. Diagnostics.

[55] Wang H., Hu D., Yao H., Chen M., Li S., Chen H., Luo J., Feng Y., Guo Y. (2019). Radiomics analysis of multiparametric MRI for the preoperative evaluation of pathological grade in bladder cancer tumors. Eur. Radiol..

[56] Li L., Zhang J., Zhe X., Tang M., Zhang L., Lei X., Zhang X. (2024). Prediction of histopathologic grades of bladder cancer with radiomics based on MRI: Comparison with traditional MRI. Urol. Oncol..

[57] Dolz J., Gopinath K., Yuan J., Lombaert H., Desrosiers C., Ben Ayed I. (2019). HyperDense-Net: A hyper-densely connected CNN for multi-modal image segmentation. IEEE Trans. Med. Imaging.

[58] Gumus K.Z., Nicolas J., Gopireddy D.R., Dolz J., Jazayeri S.B., Bandyk M. (2024). Deep learning algorithms for bladder cancer segmentation on multi-parametric MRI. Cancers.

[59] Li M., Jiang Z., Shen W., Liu H. (2022). Deep learning in bladder cancer imaging: A review. Front. Oncol..

[60] AlSaad R., Abd-Alrazaq A., Boughorbel S., Ahmed A., Renault M.A., Damseh R., Sheikh J. (2024). Multimodal large language models in health care: Applications, challenges, and future outlook. J. Med. Internet Res..

[61] Nam Y., Kim D.Y., Kyung S., Seo J., Song J.M., Kwon J., Kim J., Jo W., Park H., Sung J. (2025). Multimodal large language models in medical imaging: Current state and future directions. Korean J. Radiol..

[62] Akinci D’Antonoli T., Stanzione A., Bluethgen C., Vernuccio F., Ugga L., Klontzas M.E., Cuocolo R., Cannella R., Koçak B. (2024). Large language models in radiology: Fundamentals, applications, ethical considerations, risks, and future directions. Diagn. Interv. Radiol..

[63] Necchi A., Bandini M., Calareso G., Raggi D., Pederzoli F., Farè E., Colecchia M., Marandino L., Bianchi M., Gallina A. (2020). Multiparametric magnetic resonance imaging as a noninvasive assessment of tumor response to neoadjuvant Pembrolizumab in muscle-invasive bladder cancer: Preliminary findings from the PURE-01 study. Eur. Urol..

[64] Basile G., Bandini M., Gibb E.A., Ross J.S., Raggi D., Marandino L., Costa de Padua T., Crupi E., Colombo R., Colecchia M. (2022). Neoadjuvant Pembrolizumab and radical cystectomy in patients with muscle-invasive urothelial bladder cancer: 3-year median follow-up update of PURE-01 trial. Clin. Cancer Res..

[65] Tateo V., Basile G., Giannatempo P., de Jong J.J., Proudfoot J.A., Maiorano B.A., Cigliola A., Mercinelli C., Davicioni E., Moschini M. (2026). Updated 5-year survival results from PURE-01, a Phase 2 study of neoadjuvant Pembrolizumab followed by radical cystectomy in patients with muscle-invasive bladder cancer. Eur. Urol..

[66] Bandini M., Calareso G., Raggi D., Marandino L., Colecchia M., Gallina A., Giannatempo P., Pederzoli F., Gandaglia G., Fossati N. (2021). The value of multiparametric magnetic resonance imaging sequences to assist in the decision making of muscle-invasive bladder cancer. Eur. Urol. Oncol..

[67] O’Sullivan N.J., Temperley H.C., Corr A., Meaney J.F.M., Lonergan P.E., Kelly M.E. (2025). Current role of radiomics and radiogenomics in predicting oncological outcomes in bladder cancer. Curr. Urol..

[68] Qureshi T.A., Chen X., Xie Y., Murakami K., Sakatani T., Kita Y., Kobayashi T., Miyake M., Knott S.R.V., Li D. (2023). MRI/RNA-Seq-based radiogenomics and artificial intelligence for more accurate staging of muscle-invasive bladder cancer. Int. J. Mol. Sci..

[69] Liu S., Chen H., Zheng Z., He Y., Yao X. (2023). Development of a molecular-subtype-associated immune prognostic signature that can be recognized by MRI radiomics features in bladder cancer. Bioengineering.

